# Genome-wide identification of DCL, AGO and RDR gene families and their associated functional regulatory element analyses in sunflower (*Helianthus annuus*)

**DOI:** 10.1371/journal.pone.0286994

**Published:** 2023-06-09

**Authors:** Anamika Podder, Fee Faysal Ahmed, Md. Zahid Hasan Suman, Afsana Yeasmin Mim, Khadiza Hasan

**Affiliations:** Department of Mathematics, Faculty of Science, Jashore University of Science and Technology, Jashore, Bangladesh; Banaras Hindu University, INDIA

## Abstract

RNA interference (RNAi) regulates a variety of eukaryotic gene expressions that are engaged in response to stress, growth, and the conservation of genomic stability during developmental phases. It is also intimately connected to the post-transcriptional gene silencing (PTGS) process and chromatin modification levels. The entire process of RNA interference (RNAi) pathway gene families mediates RNA silencing. The main factors of RNA silencing are the Dicer-Like (DCL), Argonaute (AGO), and RNA-dependent RNA polymerase (RDR) gene families. To the best of our knowledge, genome-wide identification of RNAi gene families like DCL, AGO, and RDR in sunflower (*Helianthus annuus*) has not yet been studied despite being discovered in some species. So, the goal of this study is to find the RNAi gene families like DCL, AGO, and RDR in sunflower based on bioinformatics approaches. Therefore, we accomplished an inclusive *in silico* investigation for genome-wide identification of RNAi pathway gene families DCL, AGO, and RDR through bioinformatics approaches such as (sequence homogeneity, phylogenetic relationship, gene structure, chromosomal localization, PPIs, GO, sub-cellular localization). In this study, we have identified five DCL (HaDCLs), fifteen AGO (HaAGOs), and ten RDR (HaRDRs) in the sunflower genome database corresponding to the RNAi genes of model plant *Arabidopsis thaliana* based on genome-wide analysis and a phylogenetic method. The analysis of the gene structure that contains exon-intron numbers, conserved domain, and motif composition analyses for all HaDCL, HaAGO, and HaRDR gene families indicated almost homogeneity among the same gene family. The protein-protein interaction (PPI) network analysis illustrated that there exists interconnection among identified three gene families. The analysis of the Gene Ontology (GO) enrichment showed that the detected genes directly contribute to the RNA gene-silencing and were involved in crucial pathways. It was observed that the *cis*-acting regulatory components connected to the identified genes were shown to be responsive to hormone, light, stress, and other functions. That was found in HaDCL, HaAGO, and HaRDR genes associated with the development and growth of plants. Finally, we are able to provide some essential information about the components of sunflower RNA silencing through our genome-wide comparison and integrated bioinformatics analysis, which open the door for further research into the functional mechanisms of the identified genes and their regulatory elements.

## 1 Introduction

Almost all eukaryotic species contain gene silencing pathways including small RNAs (sRNAs; including 21–24 nucleotides), that have two types like miRNAs (microRNAs) and siRNAs (short interfering RNAs) [1, 2]. Also, the plant genome contains a large number of sRNA molecules [3–7]. The sRNA molecules regulation processes in plants are regulated by the proteins which encoded by three categories of RNAi gene families: Dicer-like (DCL), Argonate (AGO), and RNA dependent RNA polymerases (RDR), as well as their related regulatory elements [3–9]. DCL, AGO, and RDR genes are significant components in the sRNAs synthesis and RNAi pathways in multicellular organisms, inducing gene silencing [10, 11].

Dicer-like (DCL) proteins, which are crucial components in the miRNA and siRNA biogenesis pathways as they convert small mature RNAs from large double-stranded RNAs [12–16]. DEAD/ResIII, Helicase_C, Dicer_Dimer, PAZ, RNase III, and DSRM are the functional domains identified in DCL proteins that play a crucial role for the proteins to be effective [17]. AtDCL proteins are valuable enzymes suitable for creating both miRNAs and siRNAs [18]. They are affiliated with flowering mechanisms [19] and plant vegetative phase growth [17, 18, 20, 21].

AGO proteins are the most significant components of RNAi pathways, which play an essential and widespread role in gene repression [16]. The molecular weight of AGO proteins (approximately 90–100 kDa) is significant and Argo-N/Argo-L, PAZ, ArgoMid, and Piwi functional domains are present in AGO proteins [11, 22]. AGO proteins are mostly connected to RNA silencing mechanisms, which are also involved in transgene silencing [23], epigenetic silencing mechanisms [24], plant growth [25], and meristem maintenance [26] in various plant species.

RDR is the third most important family of RNAi associated proteins, with RdRP which is a single conserved domain. The RDR protein family is present in fungi, nematodes, and plants but it has not yet been identified in insects and vertebrates [27]. The RDR proteins share the only conserved catalytic RNA-dependent RNA polymerase (RdRP) domain.

Many commercially significant plant species have had several RNAi-related DCL, AGO, and RDR gene families which are detected and analyzed by using *in silico* technique, including 32, 20, 51, 23, 36, 25, and 38 genes in rice (*Oryza sativa*) [28], cucumber (*Cucumis sativus L*.) [29], Brassica (*Brassica sp*.) [30], barley (*Hordeum vulgare*) [31], sugarcane (*Saccharum spontaneum*) [32], sweet orange (*Citrus sinensis*) [9, 33], and foxtail millet (*Setaria italica*) [34] respectively. Also, grapevine (*Vitis vinifera*) [35], and pepper (*Capsicum annuum L*.) [36] both have 22 identified genes. In recent studies, the identified genes of banana (*Musa acuminate*) [37] was 21. In this study, *A*. *thaliana* was used as a model plant species, that contains 20 RNAi pathways that have been found and characterized in association with genes such as 4 AtDCL, 10 AtAGO, and 6 AtRDR [38–41]. Arabidopsis is commonly used as a model plant for comparative *in silico* studies [28, 30–33, 36, 37, 42].

Sunflower (*Helianthus annuus*) is widely cultivated and one of the four largest sources of vegetable oil in the world, according to Food and Agriculture Organization (FAO) in 2010 (http://www.fao.org). It is already being used as an ornamental plant and was used in ancient ceremonies [43, 44]. Sunflowers are produced in 47,347,175 tonnes per year worldwide [45]. Fast growth, lengthy flowering period, restricted water availability [46], and high yield are among the agriculturally favorable properties of sunflower. The sunflower contains valuable antimicrobial, cardiovascular, antioxidant, wound-healing, antihypertensive, and anti-inflammatory benefits found in its flavonoids, phenolic compounds, vitamins, and polyunsaturated fatty acids [47]. It protects us from many diseases like high cholesterol, cardiovascular disease, heart disease, bronchial, laryngeal, and pulmonary infections, coughs and colds, and whooping cough [48]. Despite identifying the gene family of sunflower is urgently needed but it is clear that still, no identification of the sunflower gene family is available. However, for a variety of reasons, improving sunflowers through a breeding program has proven to be a challenging task for breeders. As a result, genetic engineering techniques play a vital role in crop development. If we can identify the gene family of the sunflower, then people all across the world will be benefited from it. So, in this study, we have taken preliminary step to identify the essential gene family members into this economically significant plant.

Many plant species, including rice [49], soybean [50, 51], sugarcane [52], corn [53], strawberry [54], wheat [55], and cucumber [29], have undergone various characterization and expression analysis of target RNAi pathway genes. But this strategy has drawbacks in terms of skilled human resources, a well-equipped laboratory, a longer time, and experimental funds. Despite the significant laboratory-based investigation of RNAi genes, we can gather genome-wide information from many plant species utilizing integrated bioinformatics approaches, which might save money, labor, and time demand.

**Fig 1:**
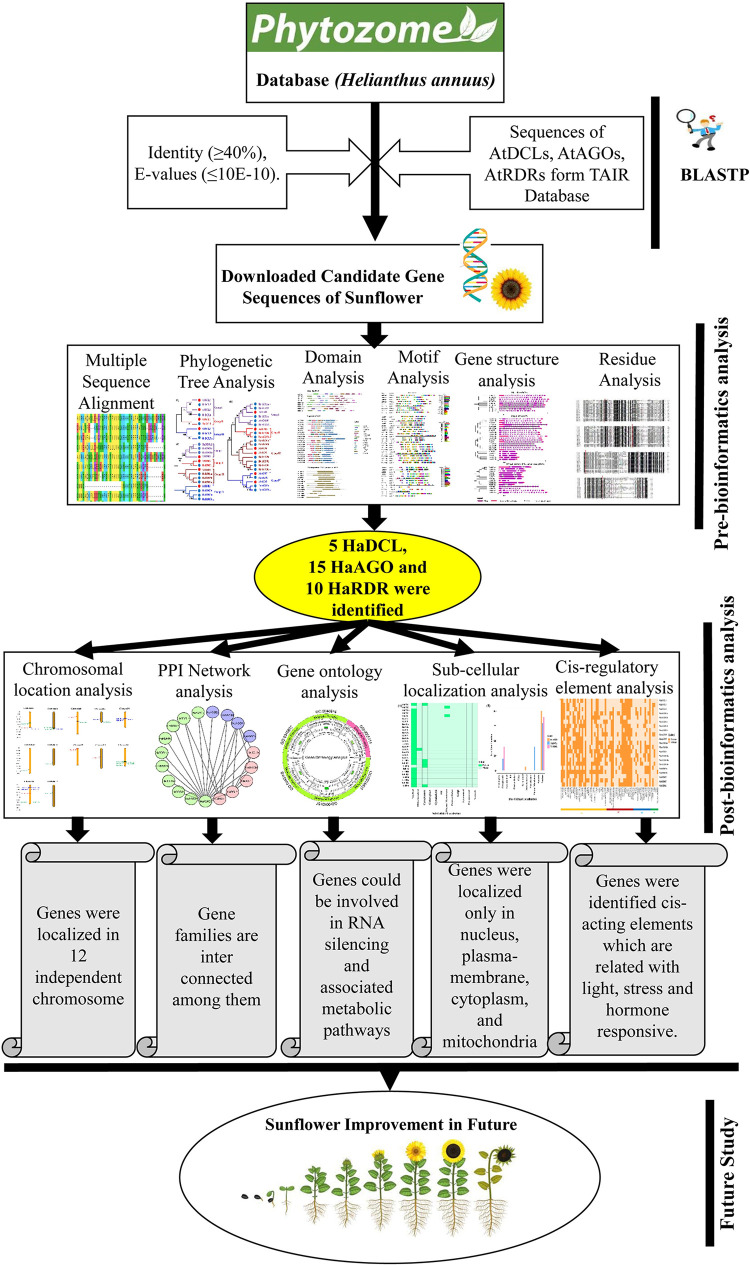
A global overview of our study.

In this study, we conducted a comprehensive *in silico* investigation using bioinformatics approaches to identify genome-wide RNAi pathway gene families DCL, AGO, and RDR. Firstly, we have used sequence similarity which is a method for characterizing newly determined sequences. Next, using a phylogenetic relationship of sunflower, we recognized and characterized the DCL, AGO, and RDR gene family including gene structure (domain, motif, and exon-intron numbers) compared with DCL, AGO, and RDR from model plant *Arabidopsis thaliana*. In addition, chromosomal localization, protein-protein interactions network, and gene ontology have been used. Finally, the subcellular localization of the testified proteins and the *cis-*acting regulatory elements connected with the *H*. *annuus* genes were also retrieved in this analysis. The complete sunflower genome sequence [56] offer us a great opportunity to identify probable RNAi pathway genes in the entire sunflower genome, which could be valuable in future sunflower improvement programs. We have described our suggested methodology graphically in Fig 1.

## 2 Materials and methods

### 2.1 Data source of DCL, AGO and RDR genes

In the beginning, we obtained protein sequences from the Phytozome database (https://phytozome.jgi.doe.gov/pz/portal.html) based on the completed *H*. *annuus* genome sequence to identify DCL, AGO, and RDR genes in sunflower (*H*. *annuus*). Additionally, we also gathered identified sRNA biogenesis protein sequences of the model plant *A*. *thaliana* (AtDCLs, AtAGOs, and AtRDRs) from a well-known database name The Arabidopsis Information Resource (TAIR) database (http://www.arabidopsis.org). Then *H*. *annuus* was searched in the Phytozome database using the Basic Local Alignment Search Tool (BLAST) program based on the Hidden Markov Model (HMM). In BLAST, HMMs are used to construct position-specific scoring matrices, which are used to score alignments between a query sequence and sequences in a reference database [56].

The extracted predicted protein sequences from *H*. *annuus* were downloaded with the essential identity percentage (≥40%) based on the BLOSUM62 matrix and the significant E-values (≤10E-10). Exclusively the initial transcript of the sequences was taken into consideration in this analysis to prevent protein sequence redundancy.

By using the *H*. *annuus* genome database, which was deposited in Phytozome we obtained genomic information such as the primary transcript, genomic length, and so on. The ExPASy- ComputepI/Mwtool (http://au.expasy.org/tools/pitool.html) was involved to estimate and forecast the molecular weight of the detected protein sequences. In this investigation, the analytically detected new DCL, AGO, and RDR genes in the *H*. *annuus* genome were given names in accordance with the nomenclature by using the phylogenetic of analogous gene family members of formerly defined *A*. *thaliana* genes.

### 2.2 Multiple sequence alignments and phylogenetic analysis

By using the Clustal-W method to conduct multiple sequence alignments of the encoded protein sequences (DCL, AGO, and RDR) of both *H*. *annuus* and *A*. *thaliana* [57] through the MEGA 11.0 software [58]. Eventually, the Neighbor-joining technique [59] was implemented to perform phylogenetic tree analysis on the aligned sequences, with 1,000 bootstrap replicates [60] performed to verify the evolutionary relationship. The Equal Input technique was used to calculate the evolutionary distances [61].

### 2.3 Conserved domain and motif analysis

Protein sequences were extracted and evaluated with the help of the protein family database (Pfam, https://pfam.xfam.org/) to look into the preserved features of the three gene families in *H*. *annuus*. The most functionally conserved domains of *H*. *annuus* (HaDCL, HaAGO, and HaRDR) that are identical to the AtDCL, AtAGO, and AtRDR proteins in *A*. *thaliana* were evaluated.

To analyze the motifs in all of the selected DCL, AGO, and RDR genes, we used the Multiple Expectation Maximization for Motif Elicitation (MEME) webserver (http://meme.sdsc.edu/meme4_3_0/cgi-bin/meme.cgi) [62]. The following parameters were defined for this purpose: (i) the best motif widths are ≥6 and ≤50; (ii) the greatest quantity of motifs is 20. Rejecting any motif that did not suit the structural domains of the proteins within every family.

### 2.4 Gene structure and chromosomal localization analysis

The online program Gene Structure Display Server (GSDS2.0: https://gsds.cbi.pku.edu.cn) was used to examine the gene structure of assessed genes [63]. Moreover, to estimate the exon-intron composition of the selected genes in *H*. *annuus*, the structures of the nominated genes were associated with the gene structure of *A*. *thaliana*. The MapGene2Chromosome V2 (http://mg2c.iask.in/mg2c v2.0/) is an online tool that was used to map the chromosomal location of identified HaDCL, HaAGO, and HaRDR genes.

### 2.5 Protein-protein interactions network analysis

Protein-protein interaction (PPI) network has emerged as a prominent focus in the field of systems biology. It analyses based on divergent structural and functional properties of proteins [64]. The PPI network was constructed by inserting predicted genes into the Search Tool for the Retrieval of Interacting Genes (STRING) database with default parameters [65]. The visualization of the PPI network was conducted using Cytoscape software [66].

### 2.6 Gene ontology and sub-cellular localization analysis

The GO analysis was completed using the online tool named Plant Transcription Factor Database (PlantTFDB, http://planttfdb.cbi.pku.edu.cn//) used to establish the association of putative RNAi-related genes with distinct clusters of molecular pathways [67]. The Fishers test and Benjamini-correction Hochberg’s were used to calculate the associated *p*-values. A statistically significant *p*-value of that GO analysis was <0.05. Using a sophisticated web server namely Plant Sub-cellular Localization Integrative Predictor (PSI), we projected the sub-cellular localization of detected HaDCL, HaAGO, and HaRDR proteins based on their protein sequences [68].

### 2.7 Promoter *cis*-acting regulatory elements (PCRE) analysis

Each RNAi gene has a start codon (ATG) in its upstream area (1.5 kb genomic sequences) that are extracted to explore the PCRE of the HaDCL, HaAGO, and HaRDR. Then, using the Plant CARE database’s (http://bioinformatics.psb.ugent.be/webtools/plantcare/html/) Signal Scan search engine, we investigated the stress-response correlated PCRE by online prediction analysis [69]. The evaluated PCRE was divided into five categories: light responsive (LR), stress responsive (SR), hormone responsive (HR), other activities (OT), and unknown function.

## 3 Results and discussion

### 3.1 *In silico* identification of RNAi-related genes in sunflower genome

By using retrieved protein sequences of *A*. *thaliana* (AtDCL, AtAGO, and AtRDR) as a query sequence to create a Hidden Markov Model (HMM) for identifying sunflower RNA silencing genes. We have identified 5, 15, and 10 genes encoding DCL (HaDCLs), AGO (HaAGOs), and RDR (HaRDRs) respectively in the sunflower genome database based on the HMM analysis. The basic information of identified RNA silencing genes is illustrated by the Table 1. That contains chromosomal location, structural features (ORF length, gene length and intron number), and protein profile (molecular weight of the encoded protein, protein length, and isoelectric point (pI)). On the basis of HMMER analysis with regards of all six types of conserved domains DEAD, Helicase_C, Dicer_dimar, PAZ, RNase III and DSRM; five DCL were identified in sunflower genome. However, this web tool provides various parameters including e-values and only statistically significant domains are shown according to e-values. The identified HaDCLs ORF ranged from 3819bp (HaDCL3a, HanXRQChr13g0398971) to 5133bp (HaDCL1, HanXRQChr01g0030961) with potentially encoded amino acids (aa) 1273 and 1711 aa (Table 1). The identified HaDCL genes has genomic length that varies from 7808 bp to 17484 bp. This genomic length was created by the HaDCL genes, HaDCL3b (HanXRQChr05g0136441) and HaDCL4 (HanXRQChr12g0356941), respectively. All of the HaDCL proteins have acidic characteristics, according to their pI values. The genome length of the selected HaAGO genes diverse from 4828 bp to 16653bp formed by the HaAGO9b (HanXRQChr14g0447541) and HaAGO10 (HanXRQChr05g0141111), in turn. The genes contain the ORF length that covering from 2610 to 4164bp (HaAGO4a and HaAGO1c) encode the testified HaAGO proteins. The potentiality of HaAGO4a and HaAGO1c are 870 aa and 1388 aa. The projected all HaAGOs proteins have higher basic characteristics, according to their pI values (pI value 8.98~9.57).

**Table 1:** Fundamental findings about the DCL, AGO, and RDR gene families in *H*. *annuus*.

Serial number	Gene name	Accession Number	Chromosomal location	ORF (bp)	Gene Length (bp)	No. of Intron	Protein		
							Molecular Weight (kD)	Protein Length (aa)	pI
HaDCL									
1	HaDCL1	HanXRQChr01g0030961	HanXRQChr01:153201827–153211244	5133	9417	18	191.99	1711	6.10
2	HaDCL2	HanXRQChr16g0511821	HanXRQChr16:82732337–82748556	4179	16219	21	158.10	1393	6.46
3	HaDCL3a	HanXRQChr13g0398971	HanXRQChr13:81072918–81082976	3819	10058	14	142.26	1273	6.39
4	HaDCL3b	HanXRQChr05g0136441	HanXRQChr05:42116273–42124081	4083	7808	17	152.86	1361	6.28
5	HaDCL4	HanXRQChr12g0356941	HanXRQChr12:7215550–7233034	4860	17484	24	182.62	1620	6.29
HaAGO									
1	HaAGO1a	HanXRQChr17g0552381	HanXRQChr17:76098482–76105643	3408	7161	22	125.95	1136	9.49
2	HaAGO1b	HanXRQChr03g0087191	HanXRQChr03:148898038–148904063	2763	6025	19	103.55	921	9.34
3	HaAGO1c	HanXRQChr06g0183501	HanXRQChr06:90434709–90444187	4164	9478	19	155.88	1388	9.45
4	HaAGO2a	HanXRQChr16g0528211	HanXRQChr16:168229186–168234290	2961	5104	2	111.26	987	9.19
5	HaAGO2b	HanXRQChr16g0528251	HanXRQChr16:168255437–168260336	3009	4899	2	112.93	1003	9.31
6	HaAGO4a	HanXRQChr03g0091711	HanXRQChr03:162762096–162769938	2610	7842	21	97.77	870	9.10
7	HaAGO4b	HanXRQChr14g0459081	HanXRQChr14:165970273–165976933	2880	6660	22	107.96	960	8.98
8	HaAGO5a	HanXRQChr11g0345791	HanXRQChr11:139263076–139271283	2919	8207	21	107.82	973	9.53
9	HaAGO5b	HanXRQChr01g0018021	HanXRQChr01:108166916–108173326	2922	6410	21	107.88	974	9.57
10	HaAGO7	HanXRQChr10g0308051	HanXRQChr10:204065672–204072169	3090	6497	3	117.15	1030	9.48
11	HaAGO9a	HanXRQChr14g0446981	HanXRQChr14:129605138–129610155	2661	5017	19	98.76	887	9.24
12	HaAGO9b	HanXRQChr14g0447541	HanXRQChr14:131448573–131453401	2727	4828	20	101.61	909	9.32
13	HaAGO9c	HanXRQChr14g0447001	HanXRQChr14:129748960–129757683	2844	8723	22	105.85	948	9.39
14	HaAGO9d	HanXRQChr14g0445731	HanXRQChr14:125146207–125152958	2781	6751	21	103.60	927	9.09
15	HaAGO10	HanXRQChr05g0141111	HanXRQChr05:92719656–92736309	3078	16653	22	116.33	1026	9.28
HaRDR									
1	HaRDR1a	HanXRQChr08g0223631	HanXRQChr08:61811073–61816820	3522	5747	5	133.94	1174	6.54
2	HaRDR1b	HanXRQChr08g0224271	HanXRQChr08:65536438–65542547	3093	6109	3	117.14	1031	6.78
3	HaRDR1c	HanXRQChr08g0223651	HanXRQChr08:61897465–61903652	3399	6187	4	129.31	1133	6.27
4	HaRDR2	HanXRQChr16g0503651	HanXRQChr16:23859541–23864650	3366	5109	3	127.43	1122	6.40
5	HaRDR3a	HanXRQChr16g0507611	HanXRQChr16:49884182–49892556	3270	8374	18	123.16	1090	7.84
6	HaRDR3b	HanXRQChr16g0503771	HanXRQChr16:24234874–24246575	4011	11701	18	149.01	1337	8.24
7	HaRDR3c	HanXRQChr16g0503801	HanXRQChr16:24293073–24296828	1638	3755	9	61.60	546	8.82
8	HaRDR6a	HanXRQChr01g0030821	HanXRQChr01:152903224–152904802	1398	1578	2	53.90	466	9.00
9	HaRDR6b	HanXRQChr03g0088731	HanXRQChr03:155915626–155921105	3606	5479	1	137.28	1202	6.38
10	HaRDR6c	HanXRQChr06g0181231	HanXRQChr06:69293995–69297357	1992	3362	2	75.47	664	6.60

According to our HMM analysis, the HaRDR gene family contains the RdRP conserved domain. The ORF length of the ten detected HaRDRs differ from 1398 bp to 4011 bp and genome length varied from 1578 to 11701 bp, which are corresponding with HaRDR6a (HanXRQChr01g0030821) and HaRDR3b (HanXRQChr16g0503771) encoded protein length 466 aa and 1377 aa (Table 1). The pI values of the HaRDRs proteins represent that the seven proteins are more likely to be basic, where the HaRDR3b, HaRDR3c, and HaRDR6a have the greatest pI value of 8.24, 8.82, and 9.00 which displayed the basic characteristics.

The pI values in various plant species are widely distributed from 1.99 to 13.96 [70]. Plant protein pI values are also important in post-translational modifications and biochemical roles in the RNAi gene family [71].

### 3.2 Multiple sequence alignment RNAi-related genes in sunflower and Arabidopsis

By using AtDCL, AtAGO, and AtRDR as reference sequences, the predicted protein sequences for HaDCL, HaAGO, and HaRDR were aligned to create the multiple sequence alignment (S1–S3 Figs). The alignment outcomes demonstrated that the RNase III catalytic sites of the estimated HaDCL proteins in the two RNase III domains at the glutamate (E), aspartate (D), aspartate (D), glutamate (E) (EDDE) position with the orthologs of AtDCLs in (S1 Fig).

The Piwi domain, first revealed in AtAGO1 [28], has three conserved metal-chelating catalytic residues (D = aspartate, D = aspartate, and H = histidine), and it is necessary for endonuclease function [72, 73]. Additionally, the positioning of 10 AtAGOs and 15 HaAGOs proteins confirmed the shared DDH trio proteins of Piwi domains for both AGOs in (S2 Fig).

Furthermore, the DxDGD catalytic motif of the RdRP conserved domain was apparent in the sequence alignment of AtRDRs with the assessed HaRDRs proteins which present in S3 Fig. The DDH/H motif was identified in the selected proteins of HaAGO1a, HaAGO1b, HaAGO5a, HaAGO5b, HaAGO7 and HaAGO10, which were analogous to AtAGO1, AtAGO5, AtAGO7 and AtAGO10 proteins respectively but HaAGO1c (DDH/P) was not similar to AtAGO1 (Table 2). The DDD/H motif was found in HaAGO2a and HaAGO2b proteins, which were similar to AtAGO2 (Table 2). The DDH/S motif was detected in HaAGO9a, HaAGO9b proteins, which were disparate to AtAGO9 proteins (DDH/R) (Table 2). Also, the DDH/A motif was perceived in HaAGO9c, HaAGO9d proteins, which were different to AtAGO9 proteins (DDH/R) (Table 2). Alternative motif DDH/P was found in HaAGO4a, HaAGO4b proteins, while the DDH/S motif was detected in AtAGO4 proteins (Table 2). In this analysis, HaAGO4 protein catalytic residues histidine (H) was altered by proline (P), which is exchanged by the fourth serine (S) residue (Table 2). Additionally, HaAGO1c protein catalytic residues histidine (H) was replaced by the proline (P) residue (Table 2). (HaAGO9a, HaAGO9b) and (HaAGO9c, HaAGO9d) protein catalytic residues histidine (H) was replaced to arginine (R), which was exchanged by the serine (S) and alanine (A) residue (Table 2). The results of motif analysis exhibited that the DDH catalytic residues structure of Piwi domains is not fully preserved in all HaAGOs proteins in sunflower.

**Table 2:** Analogy of the AGO proteins in Piwi domains between *H*. *annuus* and *A*. *thaliana*.

Serial No	*H*. *annuus r1*.*2*		*A*. *thaliana*^*b*^	
	AGO	Motif^a^	AGO	Motif^a^
1	HaAGO1a	DDH/H	AtAGO1	DDH/H
2	HaAGO1b	DDH/H	AtAGO2	DDD/H
3	HaAGO1c	DDH/P	AtAGO4	DDH/S
4	HaAGO2a	DDD/H	AtAGO5	DDH/H
5	HaAGO2b	DDD/H	AtAGO7	DDH/H
6	HaAGO4a	DDH/P	AtAGO9	DDH/R
7	HaAGO4b	DDH/P	AtAGO10	DDH/H
8	HaAGO5a	DDH/H		
9	HaAGO5b	DDH/H		
10	HaAGO7	DDH/H		
11	HaAGO9a	DDH/S		
12	HaAGO9b	DDH/S		
13	HaAGO9c	DDH/A		
14	HaAGO9d	DDH/A		
15	HaAGO10	DDH/H		

The DDH/H, DDD/H, DDH/S, and DDH/R motifs in the Piwi domain of Arabidopsis AGO proteins are required for their *in vitro* endonuclease movement [72, 74, 75]. Genetic variability in the HaAGOs inhabitants resulted in alternate of the DDH/H conserved motif of Piwi domains in the discovered HaAGOs proteins by using motif analysis. By exchanging aa residues in HaAGOs proteins could indicate a reduction in endonuclease activity, or it could indicate that the altered aa residues have important gene activities in sunflowers. To combine the reporter genes with the HaAGO genes and analyzing their appearance in a transient appearance assess utilizing model plant species like *Nicotiana tabacum* and Arabidopsis, we can confirm the gene function alterations. To realize the appropriate activities of the Piwi domain with substitutions in catalytic residues of HaAGO proteins, more gene function investigation is required.

### 3.3 Analysis of phylogenetic relationship of RNAi-related genes in sunflower and Arabidopsis

**Fig 2:**
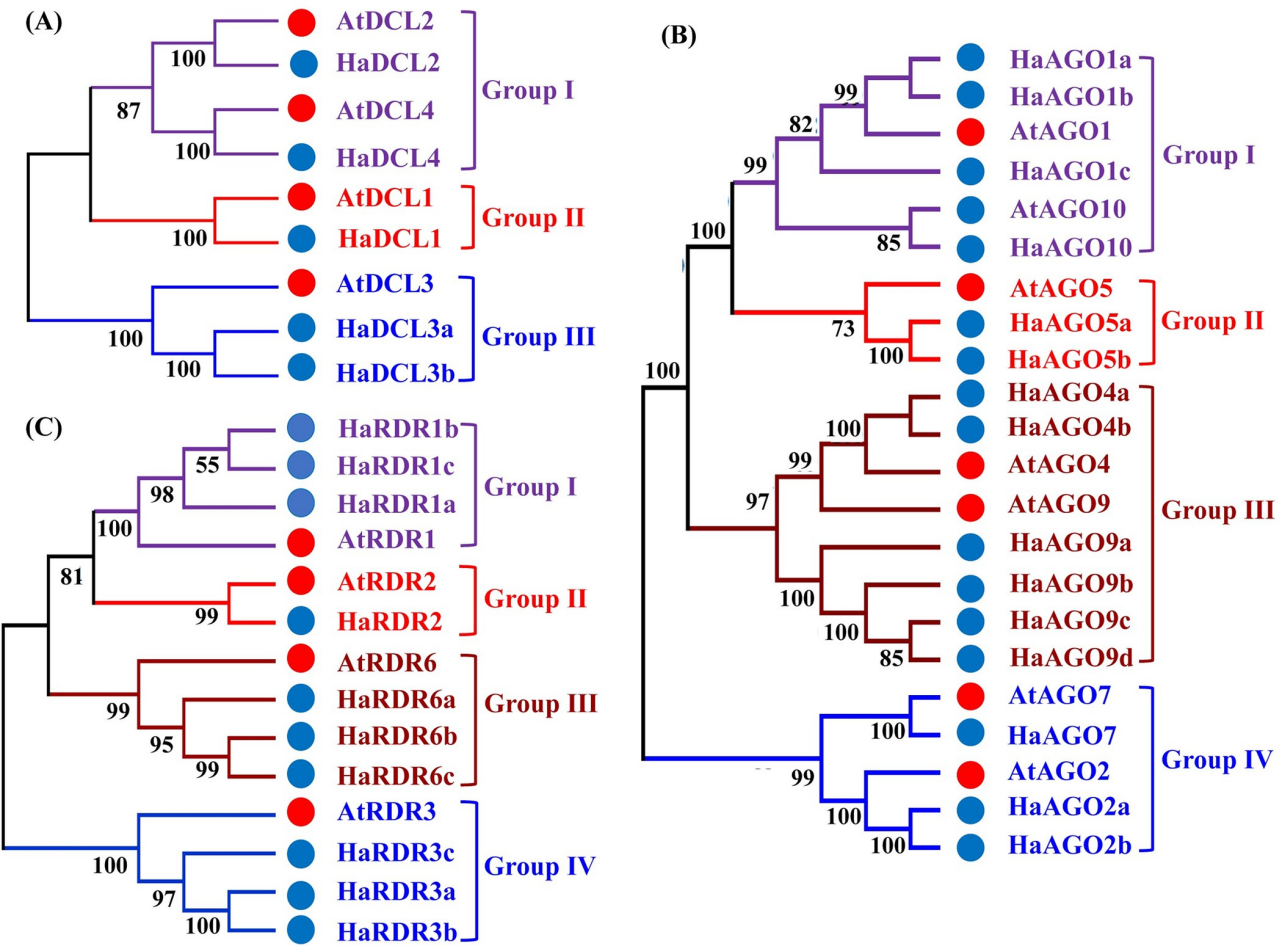
Phylogenetic tree for (A) DCL, (B) AGO, and (C) RDR proteins from *H*. *annuus* and Arabidopsis. The neighbor-joining method was used to create all of the phylogenetic trees, and the numbers at the nodes represent the percentages of bootstrap values from 1000 replications. While *A*. *thaliana* is discussed in section 2.1, *H*. *annuus* proteins are tabulated in Table 1 with their accession number and Chromosomal location. Different groups are depicted in a phylogenetic tree using different colors; *H*. *annuus* genes have been represented by blue circles, on the other hand *A*. *thaliana* genes have been presented by red circles.

A phylogenetic tree for HaDCL, HaAGO, and HaRDR proteins, as well as potential proteins from *A*. *thaliana*, was created to study the phylogenetic relationship of *H*. *annuus* RNAi associated genes (S1–S3 Datas and Fig 2A–2C). The phylogenetic tree analysis results expressed that five HaDCL proteins (HaDCL1, HaDCL2, HaDCL3a, HaDCL3b, and HaDCL4) were gathered into 3 groups (Group I-III) together with their equivalent DCL proteins in Arabidopsis with adequately-supported bootstrap values (S1 Data and Fig 2A). The AtDCL1 and AtDCL3 were significantly associated with the HaDCL1 and (HaDCL3a, HaDCL3b) proteins in Group II and Group III, correspondingly. The HaDCL1 and (HaDCL3a, HaDCL3b) comprising proteins belong to the DCL1 and DCL3 subfamilies due to their increased sequence similarity to AtDCL1 and AtDCL3, in turn. The HaDCL2 and HaDCL4 gene are clustered with AtDCL2 and AtDCL4, which was involved in Group I. Nevertheless, it was observed that HaDCL2 and HaDCL4 are DCL2 and DCL4 subfamily based on increased sequence homology with the AtDCL2 and AtDCL4 gene successively. DCL members participate in the maturation of long dsRNAs into mature sRNAs, which is an important step in the sRNA biogenesis process [12, 14].

We can confidently suppose that HaDCL1 plays a role in improvement, environmental factors, and flowering structures based on the functions of the AtDCL1 protein [1, 76]. We might predict HaDCL2 and (HaDCL3a, HaDCL3b) to be redeveloping siRNAs and trans-acting small interfering RNA (ta-siRNAs) to take part in the plant growth stage of development, resistance to disease, and flowering mechanism based on AtDCL2 and AtDCL3 functions [77, 78]. Based on the AtDCL4 function, we also can consider that HaDCL4 is taking part in ta-siRNA metabolism and plays a role in epigenetic maintenance that is mediated by RdDM during post-transcriptional silencing [21, 79].

Cluster 1 (AGO1/5/10), Cluster 2 (AGO2/7), and Cluster 3 (AGO4/9) are the three main clusters identified by the phylogenetic tree analysis of AGO genes of flowering plant species, which is identical to our work [80]. Here, 15 AGO genes had detected from *H*. *annuus* that were divided into four groups (Group I-IV) (S2 Data and Fig 2B). In group I, the name of the four sunflower proteins was HaAGO1a, HaAGO1b, HaAGO1c, and HaAGO10 were clustered with AtAGO1 and AtAGO10 respectively. The four proteins from sunflowers are classified as AGO1 subfamily because of their higher sequence similarity to AtAGO1 and AtAGO10. Group II comprises of two sunflower proteins HaAGO5a and HaAGO5b similar to the AtAGO5. Group III includes six genes from sunflower (HaAGO4a, HaAGO4b, HaAGO9a, HaAGO9b, HaAGO9c, and HaAGO9d) and two genes from Arabidopsis (AtAGO4 and AtAGO9), but (HaAGO4a, HaAGO4b) was AGO4 subfamily due to their increased sequence resemblance with the *A*. *thaliana* AGO protein AtAGO4.

Furthermore, the remaining four sunflower genes (HaAGO9a, HaAGO9b, HaAGO9c, and HaAGO9d) were involved AGO9 subfamily because they share a greater sequence likeness with the AtAGO9. Among Group IV genes, HaAGO7 and (HaAGO2a, HaAGO2b) genes cluster with AtAGO7 and AtAGO2, genes, but HaAGO7 exists in AGO7 subfamily because AtAGO7 had a greater sequence similarity. Also, HaAGO2a and HaAGO2b exists in AGO2 subfamily on the basis of higher sequence similarity with the AtAGO2. The Arabidopsis genome contained ten AGO proteins that are involved in RNA silencing [23, 81]. We can consider that HaAGO1 is associated with miRNA and transgene-silencing mechanisms based on AtAGO1’s involvement [23, 82]. We can predict that HaAGO4 is linked with endogenous siRNA activity and is essential for epigenetic silencing along with AtAGO4 function [24, 83]. On the basis of AtGAO7 and AtAGO10, we can also predict that HaAGO7 and HaAGO10 are essential for the conversion of plants from juvenile to adult stages [25] and the growth of meristem tissue [26, 84].

Additionally, four RDR gene groups (Group I-IV) were identified using phylogenetic tree analyses (S3 Data and Fig 2C). The RDR genes isolated from sunflowers have been given the following names: HaRDR1a, HaRDR1b, HaRDR1c, HaRDR2, HaRDR3a, HaRDR3b, HaRDR3c, HaRDR6a, HaRDR6b, and HaRDR6c. Group I contained three genes from sunflower (HaRDR1a, HaRDR1b and HaRDR1c) and gene from Arabidopsis AtRDR1, but HaRDR1a, HaRDR1b and HaRDR1c belongs to the RDR1 subfamily based on increased sequence resemblance with the *A*. *thaliana* RDR protein AtRDR1. Group II comprises of sunflower protein HaRDR2 similarity with the AtRDR2. Moreover, the HaRDR2 protein belongs to the RDR2 subfamily since its sequence is comparable to that of the *A*. *thaliana* RDR protein AtRDR2. According to their similarity in sequence to AtRDR6, the HaRDR6a, HaRDR6b, and HaRDR6c proteins were grouped (Group III) with that protein and constitute the RDR6 subfamily. Group IV contains 3 genes from sunflower (HaRDR3a, HaRDR3b, and HaRDR3c) and gene from Arabidopsis AtRDR3, but HaRDR3a, HaRDR3b and HaRDR3c are RDR3 subfamily on the basis of higher sequence similarity with the *A*. *thaliana* RDR protein AtRDR3. RDR proteins are able to create dsRNAs from sRNA in order to start an RNA silencing signal [85, 86]. According to the AtRDR1 function, we can estimate that HaRDR1a, HaRDR1b, and HaRDR1c may be activated by salicylic acid and serve as important elements of the RNA silencing pathway, and transgene silencing in several kinds of plants [41, 87, 88]. We can claim that HaRDR2 may be engaged in producing siRNA and connected to chromatin modification in regard to AtRDR2 function [20, 89]. The HaRDR6a, HaRDR6b, and HaRDR6c should be able to create the ta-siRNA precursor and aid in antiviral protection by degrading RNA molecules, as stated by the AtRDR6 function [90].

We observed that the entire sunflower genome lacked HaAGO3, HaAGO6, HaAGO8, HaAGO9, HaRDR4, and HaRDR5. These findings demonstrate their functional variability, which is beneficial for further sunflower development.

### 3.4 Conserved domain and motif analysis of RNAi-related genes in sunflower and Arabidopsis

**Fig 3:**
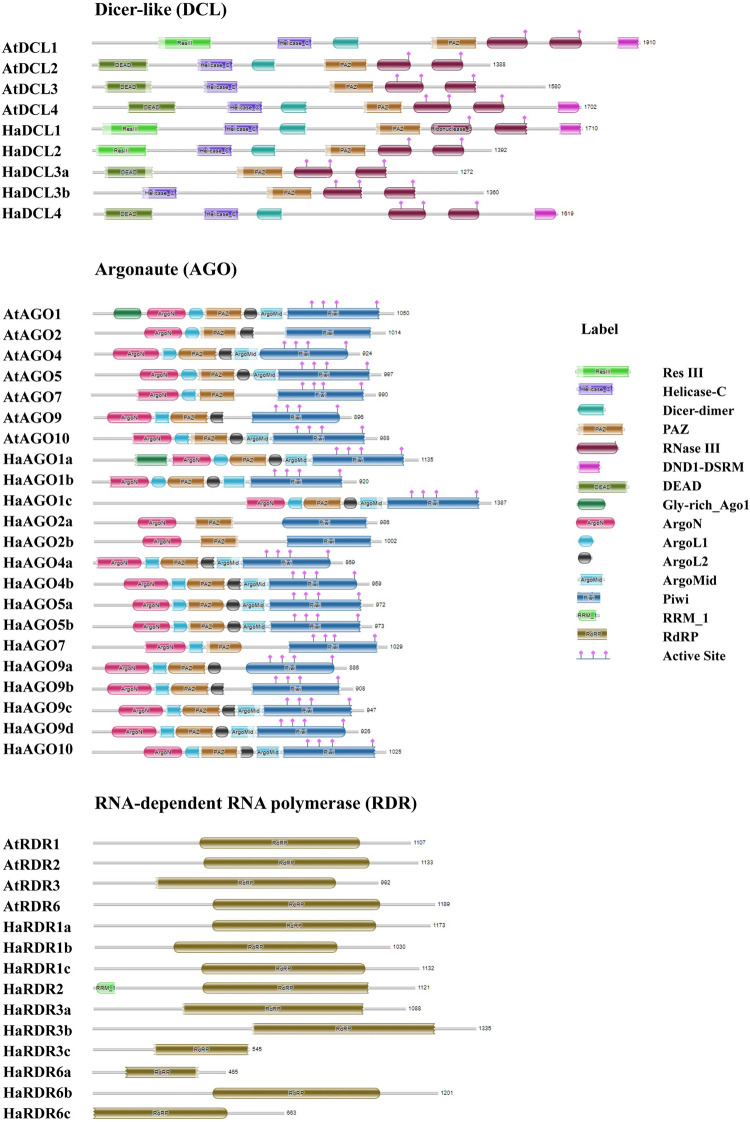
The Pfam database information was used to infer the conserved domains of the estimated HaDCL, HaAGO, and HaRDR proteins.

The most of functional domains in the DCL, AGO, and RDR families from sunflower and Arabidopsis are significantly conserved, according to the domain analysis (Fig 3). The HaDCL proteins included each of the necessary conserved domains (Fig 3). HaDCL1 and HaDCL2 proteins were similar to AtDCL1 and AtDCL2 respectively. Also, HaDCL3a and HaDCL3b were closely paralogs to AtDCL3 but Helicase_C and DEAD domain were not present in HaDCL3a and HaDCL3b successively. Only the PAZ domain was not found in the HaDCL4 protein. Natural mutation or genetic diversity or molecular evolution may be the main cause of the differential results. Because of this, the results may reflect reduced endonuclease activity or play significant gene functions in sunflower which may different from the Arabidopsis. Therefore, further gene function analysis of the *H*. *annuus* proteins in the wet lab is needed to realize the exact activity of the protein. Previous research findings indicated that these predicted domains were critical for plant protein function [21, 91, 92]. It was previously observed that the simultaneous event of two DCL genes was essential in plant defense against viral infection [93].

The PAZ domain at the N-terminus and the Piwi domain at the C-terminus are the two domains that distinguish AGO proteins [10, 22, 94–96]. All HaAGO proteins were predicted to have both PAZ and Piwi domains. The functional domains of the anticipated AGOs were found to be identical to those of the previously discovered AtAGO proteins [73]. The PAZ and Piwi domains of AGOs have been shown to be important in RNase activity in previous research [22, 97, 98]. Among the HaAGO family, (HaAGO4a and HaAGO4b), (HaAGO5a and HaAGO5b), HaAGO7, and HaAGO10 showed the domain similarity with their paralogs AtAGO4, AtAGO5, AtAGO7, and AtAGO10 respectively. The Gly-rich Ago1 domain projected in HaAGO1a proteins was similar to AtAGO1 but it was not present in HaAGO1b and HaAGO1c. To improve AGO protein stimulation for the RNA silencing process, the Gly-rich Ago1 domain coordinates interact with the ribosome [99]. The HaAGO5 protein may play a significant role in numerous sunflower stressors, which will be explained by future classification of this protein.

RDRs are engaged in the beginning of a novel RNAi silencing phase by synthesizing dsRNAs from single-stranded RNAs (ssRNAs). RDR proteins contain a catalytic β’ subunit of RdRP motif and a single conserved domain of RdRP [28, 100–102].

**Fig 4:**
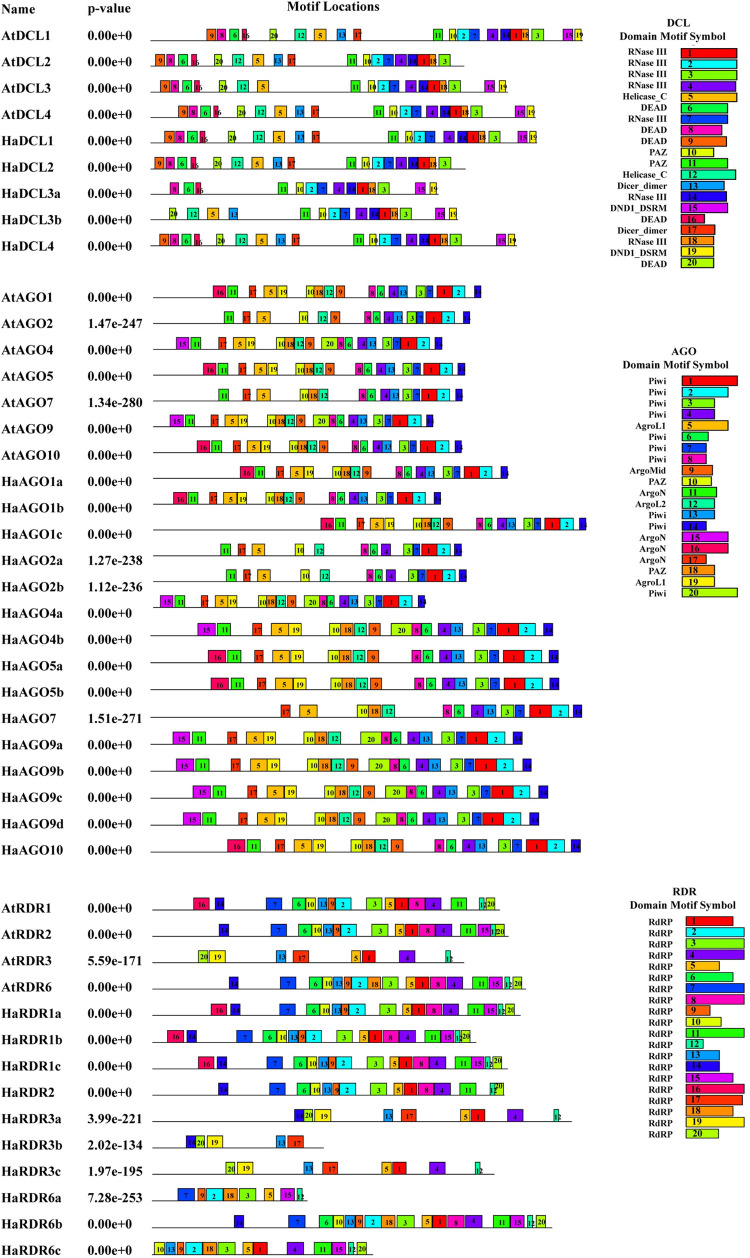
Using MEME-suite, the conserved motifs of the proposed HaDCL, HaAGO, and HaRDR protein families are shown (a maximum of 20 motifs are highlighted). In the predicted protein domains, each color corresponds to a distinct motif.

In our study, we estimated the usual RdRP domain in all HaRDR proteins, which demonstrated a likeness to the RdRP conserved region in AtRDRs. The ability to predict motifs in a protein sequence provides crucial information for identifying their functional regulatory roles in gene expression [62]. Typical motifs that are evenly spaced were predicted and preserved in HaDCL, HaAGO, and HaRDR proteins (Fig 4).

In both the HaDCL and AtDCL proteins, we counted up to 20 motifs. HaDCL1 may show strong functional similarity to AtDCL1. But the paralogs AtDCL1 and AtDCL4 shared 20 motifs with HaDCL1 and HaDCL4, respectively. There were 18 motifs in the HaDCL2 that are paralogous to the AtDCL2 motifs. While AtDCL3 proteins have 18 motifs, HaDCL3a and HaDCL3b only had 14 and 15 motifs respectively.

We were unable to locate motif (9, 20) and (5, 12) of DEAD and Helicase_C domain in HaDCL3a. Furthermore, when compared to the AtDCL4 protein, HaDCL4 lacked the DEAD domain motifs 6, 8, 9, and 16. There may be structural differences between Arabidopsis and sunflower as a result of the missing of DEAD motifs in both HaDCL3a and HaDCL3b.

On the contrary, we also estimated that HaAGOs would have a maximum of 20 motifs. We found 19 motifs in HaAGO4 (HaAGO4a and HaAGO4b), and HaAGO9 (HaAGO9b, HaAGO9c, and HaAGO9d), which displayed better conservation as compared to their paralogs AtAGO4 and AtAGO9s. Also, HaAGO9a that contains 18 motifs was closely homologous with AtAGO9. These findings indicate the high homology of the HaAGO4 and HaAGO9 proteins, which will result in similar functional properties to those of AtAGO4 and AtAGO9s. The HaAGO1 (HaAGO1a, HaAGO1b, and HaAGO1c), HaAGO5 (HaAGO5a, HaAGO5b), and HaAGO10 comprises of 18 motifs that are similar to the paralogs AtAGO1, AtAGO5, and AtAGO10 respectively. Furthermore, there was a little motif diversity between both the Arabidopsis and sunflower in AGO2 and AGO7. The motif 9 of ArgoMid, 13 of Piwi domain was not found in HaAGO2a, HaAGO2b but exist in ATAGO2. When compared to AtAGOs, the motif heterogeneity in HaAGOs supports a variety of biological functions in the RNAi silencing process.

In the HaRDR family, we predicted 5–17 RdRP conserved motifs. When compared to their paralogs AtRDR1, the HaRDR1a, HaRDR1b, and HaRDR1c families had a motif that was highly similar. These findings point to a close functional resemblance that will need to be examined further in the future by wet-lab characterization. Besides, other HaRDRs proteins, HaRDR3a, HaRDR3b, and HaRDR3c proteins contained 9, 5, and 8 conserved motifs, respectively, which displayed that HaRDR3c was similar to paralog AtRDR3 but the other two proteins discrepancy with their paralogs AtRDR3. Besides, HaRDR2, HaRDR6a, HaRDR6b, and HaRDR6c proteins contain 15, 8, 17, and 13 motifs showing that HaRDR6b was similar with paralog AtRDR6 but the other proteins mismatch between their paralogs AtRDR2 and AtRDR6. The motif 15 of RdRP domain was missing from AtRDR1 proteins but present in AtRDR1a, AtRDR1b, and AtRDR1c. The majority of the conserved domains and motifs were well conserved in HaDCL, HaAGO, and HaRDR proteins, according to the analysis of conserved domains and motifs. All three proteins have different distributions of conserved domains and motifs, indicating that they could have different functions.

### 3.5 Analysis of gene structure and chromosomal location

**Fig 5:**
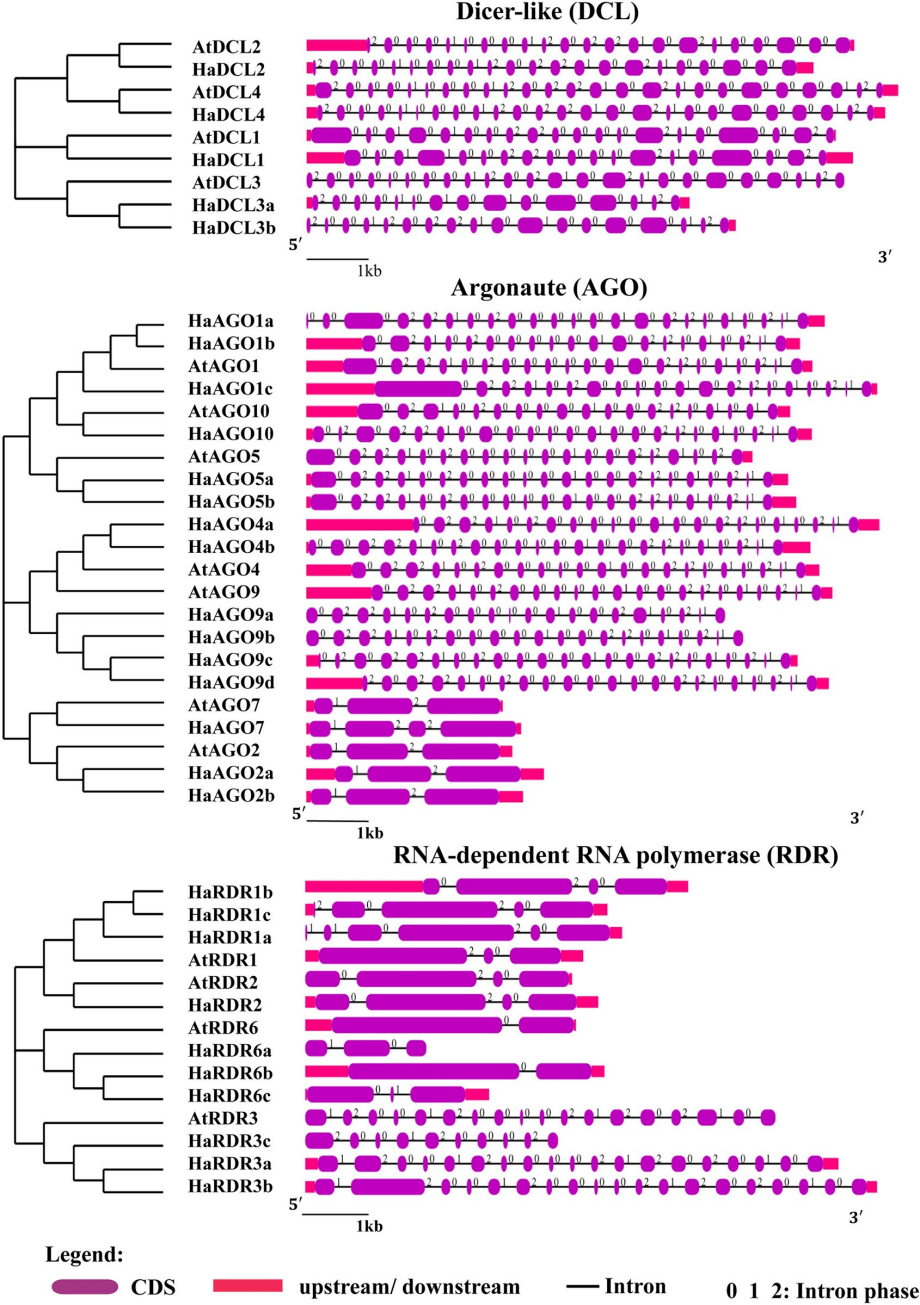
Gene formation of the projected HaDCL, HaAGO, and HaRDR proteins in *H*. *annuus* with Arabidopsis by using Gene Structure Display Server (GSDS 2.0, http://gsds.cbi.pku.edu.cn/index.php) [63].

According to the gene structure study, the predicted HaDCL, HaAGO, and HaRDR genes have well-conserved gene structures that are identical to the reference Arabidopsis genes (Fig 5). Except for HaDCL3a, the exon-intron numbers of projected HaDCLs were larger than those of AtDCLs (Fig 5 and Table 1). The HaDCLs intron numbers [12–17, 20, 22] stated similarity with AtDCLs. Out of 15 HaAGO genes, 12 HaAGO genes exhibited 19–22 intron, except for HaAGO2a, HaAGO2b, and HaAGO7 that has 2, 2, and 3 introns respectfully (Fig 5). The HaAGOs displayed introns with maximum variable numbers of (2–23), which were very comparable to the AtAGOs gene structures. However, eight HaRDR genes, with the exception of HaRDR3a and HaRDR3b, showed 2–10 numbers of introns in their gene structures, which were identical to those of the AtRDR genes (Fig 5). HaDCL, HaAGO, and HaRDR gene structures shared a higher level of similarity with their orthologs Arabidopsis, indicating that these genes have key roles in the RNAi pathway that are quite similar to one another.

**Fig 6:**
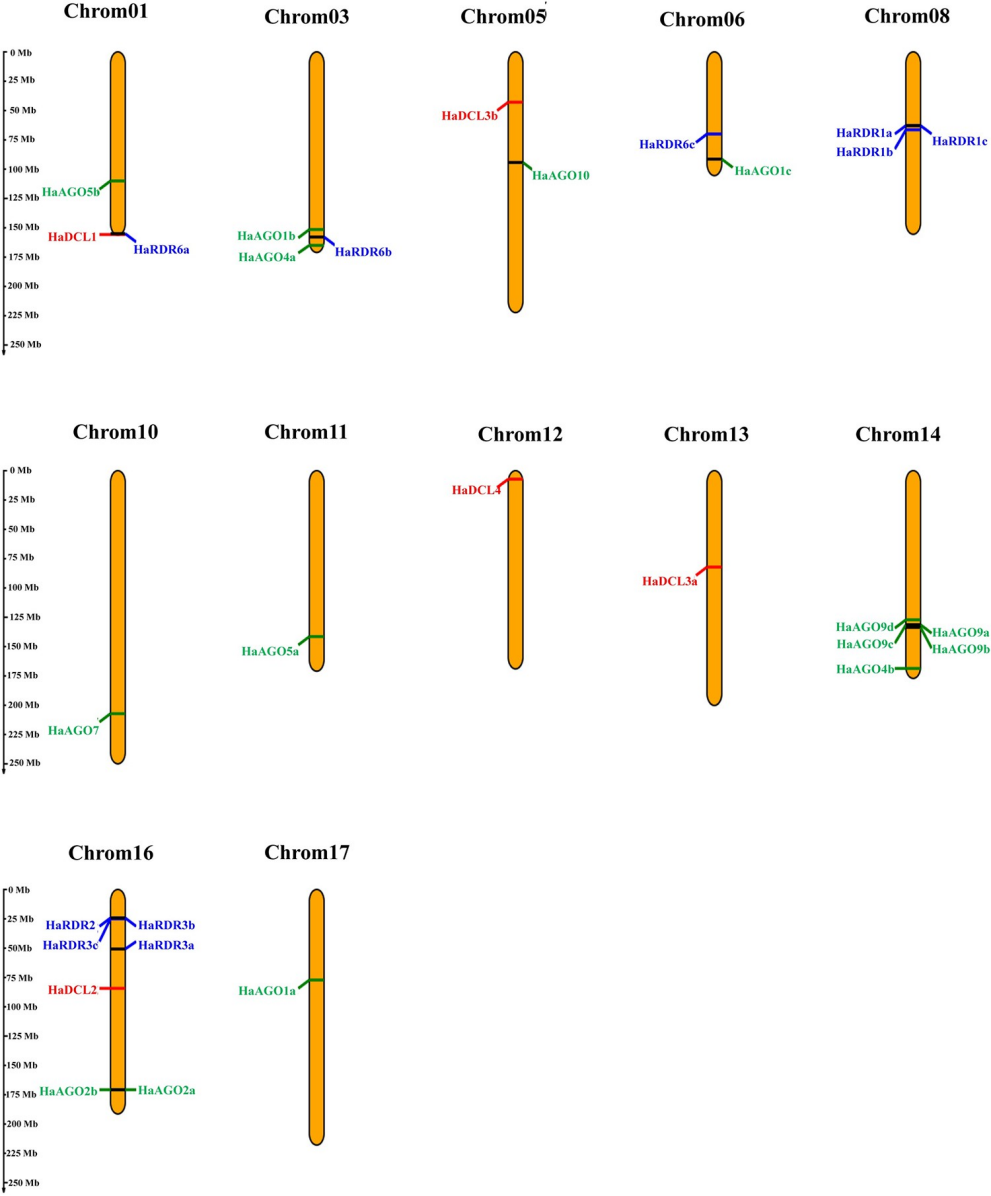
The genomic location of the predicted HaDCL, HaAGO, and HaRDR genes. On the left, a scale is presented to show the chromosomal length. ChrUn denotes a chromosome that is unknown.

The findings of the analysis of chromosomal localization showed that the mapped copies of the HaDCL, HaAGO, and HaRDR genes were distributed among 30 distinct scaffolds on the 12 separate chromosomes (Fig 6, Table 1).

Within the 12 chromosomes of the sunflower genome, the HaDCLs, HaAGOs, and HaRDRs each had a distinct scaffold position. The five HaDCL genes, HaDCL1, HaDCL2, HaDCL3a, HaDCL3b, and HaDCL4, were located in the five independent chromosomes, i.e., chromosome 1 (HaDCL1), chromosome 5 (HaDCL3b), chromosome 12 (HaDCL4), chromosome 13 (HaDCL3a), and chromosome 16 (HaDCL2). HaAGO genes are positioned in the chromosome 1 (HaAGO5b), chromosome 3 (HaAGO1b, HaAGO4a), chromosome 5 (HaAGO10), chromosome 6 (HaAGO1c), chromosome 10 (HaAGO7), chromosome 11 (HaAGO5a), chromosome 14 (HaAGO4b, HaAGO9a, HaAGO9b, HaAGO9c, HaAGO9d), chromosome 16 (HaAGO2a, HaAGO2b), and chromosome 17 (HaAGO1a). Besides this, HaRDR genes appear in chromosome 1 (HaRDR6a), chromosome 3 (HaRDR6b), chromosome 6 (HaRDR6c), chromosome 8 (HaRDR1a, HaRDR1b, HaRDR1c), and chromosome 16 (HaRDR2, HaRDR3a, HaRDR3b, HaRDR3c). Gene pairs ((HaRDR1a, HaRDR1c), HaRDR1b), ((HaAGO9a, HaAGO9b, HaAGO9c), HaAO9d) and ((HaRDR2, HaRDR3b, HaRDR3c), HaRDR3a) were closely located to each other in the genomic location of chromosomes 8, 14 and 16, in turn. These findings shown that, because of their close genomic location, these two genes may be expressed in a variety of ways, and that more research should be done under different stress circumstances.

### 3.6 PPI network analysis of RNAi-related genes in sunflower

**Fig 7:**
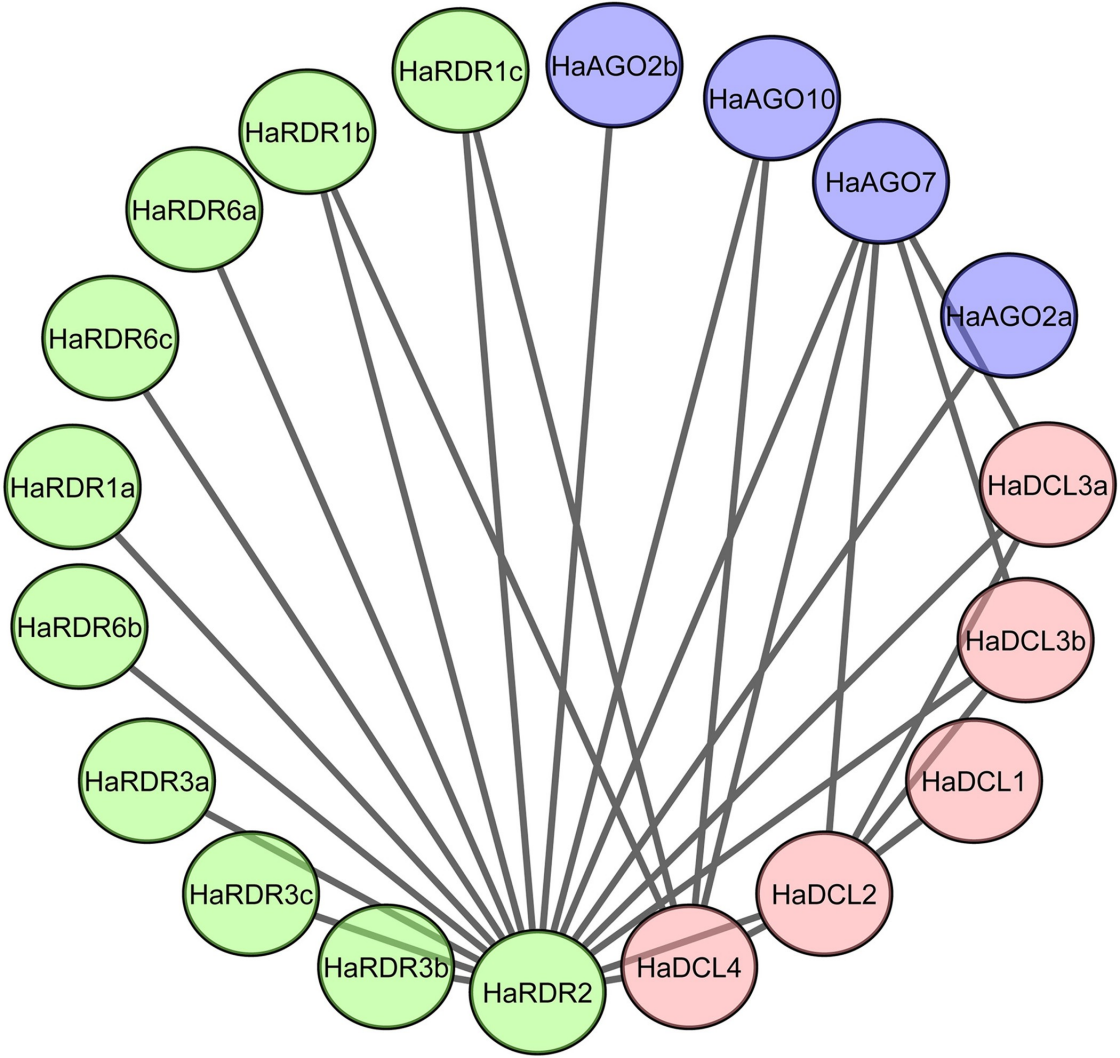
Protein-protein interaction (PPI) network of identified RNAi-related genes based on STRING database.

In this study, we employed PPI analysis to investigate the connections among thirty genes that we identified in sunflower. Among the identified genes, 19 genes were visualized using PPI network analysis, as depicted in Fig 7. Moreover, our analysis demonstrated a reciprocal connection between RDR to AGO and DCL, DCL to AGO and RDR, and AGO to DCL and RDR. This information indicating that these three gene families are interconnected with each other akin to gene silencing mechanisms. Hence, this analysis holds significant relevance in the context of gene silencing. In Fig 7, we observed that HaRDR2 exhibited the highest number of connections, being linked to 17 genes. On the other hand, HaAGO7 demonstrated connections with 5 genes, while HaDCL4 connected with 4 genes.

### 3.7 Analysis of gene ontology of RNAi-related genes in sunflower

**Fig 8:**
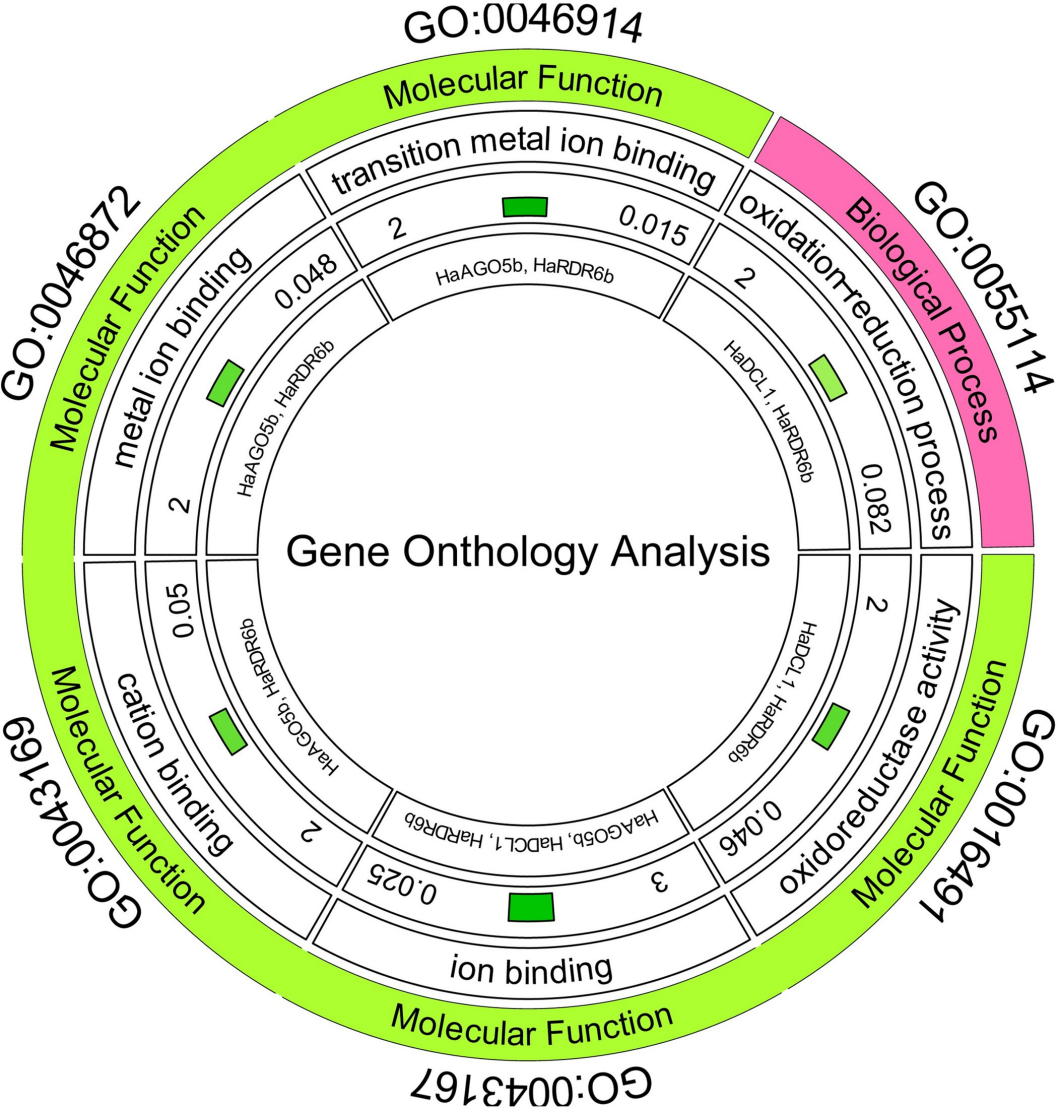
The circle graph for the predicted GO terms relating to the expected RNAi genes is shown for biological process and molecular function. Here the first track to fifth track of the figure represents GO ID, type of GO, name of activity, number of a gene family, *p*-value and corresponding significant color concerning the *p*-value, name of the different gene families respectively.

We thoroughly predicted the biological and molecular functions of the potential RNAi-related genes using GO analysis (Fig 8). The circle graph for the predicted GO terms relating to the expected RNAi genes is shown the first track of the figure depicts several gene ontologies, then the next track represents a biological and molecular function. The third track described the activity of these two functions such as oxidation-reduction process, oxidoreductase activity, cation binding activity, ion binding activity, metal ion binding activity, and transition metal ion activity. Furthermore, the fourth track represented a number of gene families, *p*-value, and corresponding significands color concerning the *p*-value, which color was a depth the *p*-value is more significant. Finally, the last track indicated the name of the different gene families.

The findings of the GO analysis showed that 2 genes in biological function (HaDCL1 and HaRDR6b) got involved in gene silencing (GO:0055114; *p*-value: 0.082) that function is an oxidation-reduction process. The relationship between RNAi and sunflowers, which would be implicated in the oxidation of mRNA, was identified by a thorough analysis of probable genes. It could help to explain the variations in apparent gene transcription that occur during seed ripening [103]. We identified 2 genes (HaDCL1 and HaRDR6b) related to oxidoreductase activity (GO:0016491; *p*-value: 0.046). Moreover, we also identified 2 genes (HaAGO5b and HaRDR6b) which were connected with cation binding activities (GO:0043169; *p*-value: 0.05). In light of the findings of the GO analysis, two RNAi genes (HaAGO5b and HaRDR6b) displayed the transition metal ion binding activity among the predicted 30 RNAi associated genes (GO:0046914; *p*-value: 0.015). The predicted 2 genes (HaAGO5b and HaRDR6b) are involved in molecular function with metal ion binding activities (GO:0046872; *p*-value: 0.048), 3 genes in molecular function (HaDCL1, HaAGO5b, and HaRDR6b) are related to ion binding (GO:0043167; *p*-value: 0.025). These findings suggested that in sunflowers, a vast number of RNAi genes are linked to several biological and molecular processes. Therefore, the investigation of gene ontologies may be directly or indirectly related to the development of the sunflower gene.

### 3.8 Sub-cellular localization of RNAi-related genes in sunflower

**Fig 9:**
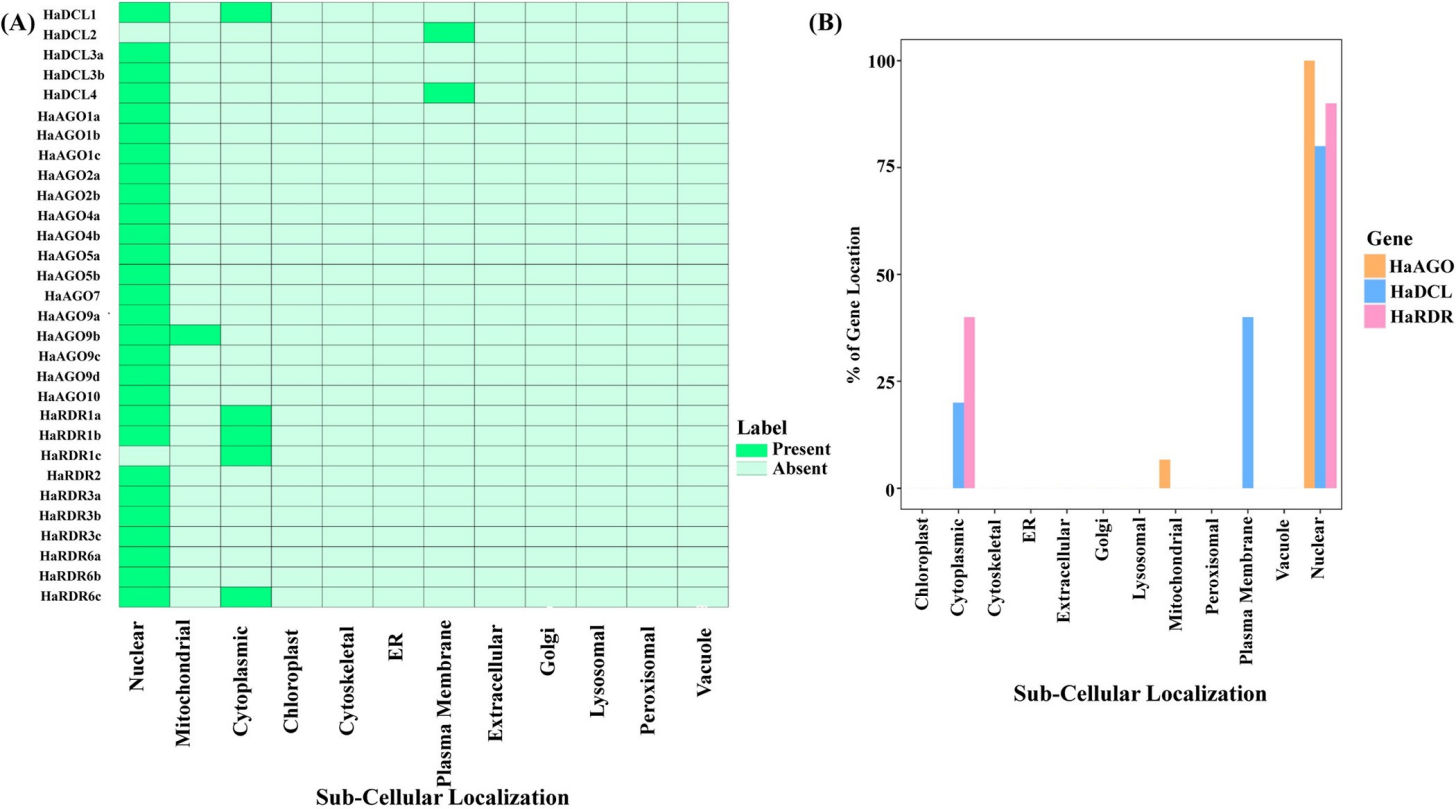
Sub-cellular localization analysis for the (A) HaDCL, HaAGO, and HaRDR proteins. (B) The percentage of protein was visible in several cellular organelles.

The subcellular localization of particular proteins is linked to the biological processes of eukaryotic cells. Understanding the functional roles of proteins at the cellular level is aided by their cellular location [104, 105]. The detected HaDCL, HaAGO, and HaRDR proteins were only found in the nucleus, plasma-membrane, cytoplasm, and mitochondria, according to subcellular localization analysis (Fig 9A). The HaDCL1 protein was found to be distributed throughout the nucleus and cytoplasm. Moreover, the HaDCL2 protein was found exclusively in the plasma-membrane. On the other hand, HaDCL3a and HaDCL3b proteins appeared only in the plasma-membrane. HaDCL4 proteins can also be found in the plasma membrane and nucleus. Unexpectedly, all HaAGO proteins were found only in the nucleus but among them HaAGO9b proteins occur in the nucleus and mitochondria. Among the ten HaRDR proteins 6 proteins (HaRDR2, HaRDR3a, HaRDR3b, HaRDR3c, HaRDR6a, and HaRDR6b) were present in the nucleus only. HaRDR1a, HaRDR1b, and HaRDR6c proteins were predicted in both nucleus and cytoplasm organelles. Only the cytoplasm and nucleus contain the HaRDR1c protein. A computational method for sub-cellular localization of DCL, AGO, and RDR proteins in *H*. *annuus* was undertaken based on protein sequences, and they were predicted in cell organelles such as the nucleus, cytoplasm, plasma membrane, mitochondria, and plastid [9].

Furthermore, earlier research has indicated that the Arabidopsis RNAi proteins AGO4 and DCL3 were co-located in the nucleus and led the RNAi silencing process [106]. The functions of the identified DCL, AGO, and RDR proteins in the RNAi pathway were revealed by our computationally-based prediction. According to the percentage-based graph (Fig 9B), HaAGO is entirely found in the nucleus and just 6.67% in the mitochondria, while HaDCL is 80% in the nucleus, 20% in the cytoplasm, and 40% in the plasma membrane. HaRDR is similarly distributed, with 90% in the nucleus and 40% in the cytoplasm.

### 3.9 Prediction of *cis*-acting regulatory elements of RNAi-related genes in sunflower

**Fig 10:**
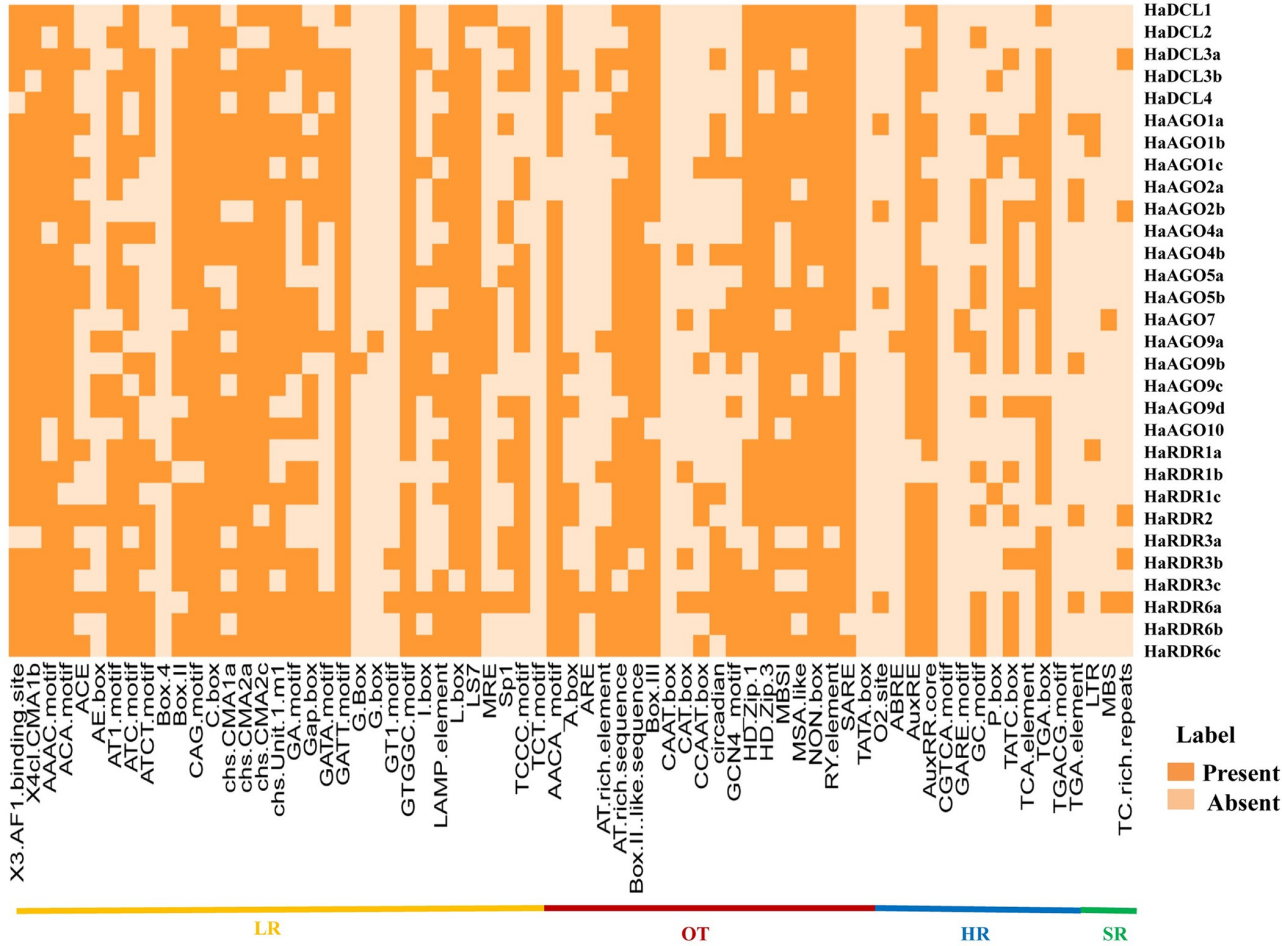
The projected HaDCLs, HaAGOs, and HaRDRs genes each have CAREs in their upstream promoter region. The element’s presence together with the related genes are shown by the deep hue.

The non-coding DNA sequences are known as "*cis*-regulatory elements (CAREs)" that contain a short motif (5–20 bp) where transcription factors (TFs) may adhere to particularly targeted genes to start transcription and control gene regulation [107, 108]. Due to the advent of high-throughput genome sequencing technology, a substantial volume of sequencing data on commercially significant crops is being produced annually [109]. As a result, we may use integrated bioinformatics tools to quickly browse the database and analyze for functional regulatory regions with specific gene functions inside their DNA sequence (usually the promoter and enhancer region). By using CAREs analysis, we have identified the numerous key motifs, their functional significance, and the variety of the projected DCL, AGO, and RDR genes in sunflowers (S4 Data and Fig 10). The light response, which takes place in the leaf tissue of sunflowers, is influenced by photosynthesis, a vital physiological parameter [110].

When compared to the other motifs, the LR motifs were more prevalent in the upstream regulatory regions of RNAi genes. The vast majority of the identified RNAi genes in sunflower contained the following motifs: LR motifs, 3-AF1 binding site, AAAC motif, ACA motif, AT1 motif, ATC-motif, ATCT-motif, box-II, chs unit1m1, GA motif, GATT-motif, GTGGC motif, LAMP element, L-box, and LS7. In addition, some additional significant LR motifs were identified in this analysis, including 4c1-CMA1b, ACE, AE-box, Box-4, CAG- motif, C-box, chs-CMA1a, chs-CMA2a, chs-CMA2c, Gap box, GATA motif, G-box, G box, GT1 motif, I-box, MRE, Sp1, TCCC motif, and TCT motif which have been shared their CAREs by RNAi genes in sunflower. Earlier research has revealed that these hypothesized LR-related motifs play a crucial part in the light response of several species [111–114]. In addition, an *in-silico* investigation of the DCL, AGO, and RDR gene families in *H*. *annuus* indicated nearly identical LR motifs, which were hypothesized to be important in the photosynthetic procedure in the leaves [9]. The photosynthetic process in sunflower leaves may therefore be significantly influenced by suggested motifs connected to LR, potentially enhancing grain amount and quality.

We have identified AACA-motif, A box, ARE, AT-rich element, AT-rich sequence, BoxII-like sequence, Box III, CAAT-box, CAT-box, CCAAT-box, circadian, GCN4-motif, HD Zip1, HD Zip3, MBSI, MSA-like, Non-box, RY element, SARE, TATA box strongly correlated with numerous biological processes in plants are highly shared by several RNAi genes predicted in sunflower (Fig 10). Plant hormones, also known as plant growth regulators, have a regulatory role in plant growth and development, either individually or coordinately [115–118]. These plant growth regulators play crucial roles in seed germination, plant development, and metabolism activities [107, 119–123]. We also predicted various plant HR motifs such as O2 site, ABRE, AuxRE, Aux RR core, CGTCA-motif, GARE-motif, GC-motif, P-box, TATC-box, TCA-element, TGA-box, TGACG-motif, TGA-element, which are common to the majority of RNAi genes found in sunflower. The presence of HR-related motifs in sunflowers shows that they play a significant biological function. Moreover, MBS, LTR, and TC-rich repeats were shared with several predicted RNAi genes. The role of TC-rich repeats LTR elements, MBS, and DRE as SR motifs in various plant species has been determined by various research groups [9,124–126]. Additionally, this investigation identified some previously unidentified CAREs. Common CAREs found in the probable RNAi gene family of the sunflower will be crucial in giving details about their functional roles in growing plants, reproduction, and defense against microbial infection.

## 4 Conclusion

In this study, we used an integrative bioinformatics strategy to identify RNAi pathway genes in the sunflower genome. In this analysis, we have identified that the sunflower genome contains 5 DCL, 15 AGO, and 10 RDR genes. The phylogenetic analysis revealed that all RNAi gene subfamilies have maintained their highest evolutionary relationship with sunflower and Arabidopsis RNAi genes. Their functional resemblance to sunflower and Arabidopsis RNAi genes was revealed by their conserved domain, motif, and gene structure. The majority of the identified HaDCL, HaAGO, and HaRDR genes are linked to significant biological functions such as RNA silencing, pathogen defense, and metabolic activity, according to GO analysis. Also, the PPI analysis revealed a relationship among the gene families, indicating their interdependence on each other. Furthermore, the subcellular localization study confirmed the reported genes and proteins as critical factors in the RNAi process in *H*. *annuus*, and most of the HaDCL, HaAGO, and HaRDR proteins were prevalent in the cytoplasm and nucleus. The TFs of putative HaDCL, HaAGO, and HaRDR genes were expected to bind the studied CAREs connected with LR, SR, and HR. As a result, our outcomes will give useful information on the sunflower genome’s DCL, AGO, and RDR genes, which could be useful for cloning and classification of sunflower RNAi genes in wet lab conditions, and these genes will be improved for use in breeding efforts for this commercially significant crop species. Furthermore, these may serve as a foundation for additional functional investigation of RNAi pathway genes in *H*. *annuus* to better understand their roles in growth, development, and disease resistance, as well as increase sweet orange production and quality.

## Supporting information

S1 Data Full-length protein sequences of DCL gene families of *A*. *thaliana* and *H*. *annuus* plant species. (TXT)

S2 Data Full-length protein sequences of AGO gene families of *A*. *thaliana* and *H*. *annuus* plant species. (TXT)

S3 Data Full-length protein sequences of RDR gene families of *A*. *thaliana* and *H*. *annuus* plant species. (TXT)

S4 Data The predicted *cis*-acting regulatory elements of the upstream promoter region (1.5 kb genomic sequences) of RNAi gene families in sunflower. (XLSX)

S1 Fig The aa sequences of the H. annuus and Arabidopsis AGO proteins were aligned numerous times using the Clustal-W tool in MEGA 11. The investigation included the RNase III domains (RIBOc I and II), the Piwi domain, and the RdRP conserved domain. The conserved positions of the two RNase III domains at the glutamate (E), aspartate (D), glutamate (E), aspartate (D) (EDDE) position are indicated by downward red arrows.
(TIF)

S2 Fig Piwi domain of the aa sequences of H. annuus and Arabidopsis AGO proteins based on Clustal-W program in MEGA 11. The conserved H798 locations and the conserved DDH triad of the Piwi domain are indicated by the descending red arrows.
(TIF)

S3 Fig RdRP conserved domain of the aa sequences of *H*. *annuus* and Arabidopsis RDR proteins by Clustal-W program in MEGA 11. The red box indicates the conserved DxDGD catalytic motif.
(TIF)
