# Supplementary figures and images for: Genome-wide identification of DCL, AGO and RDR gene families and their associated functional regulatory element analyses in sunflower (Helianthus annuus)
Source: PLoS One. 2023 Jun 9;18(6):e0286994. doi: 10.1371/journal.pone.0286994 (PMC10256174; doi:10.1371/journal.pone.0286994)

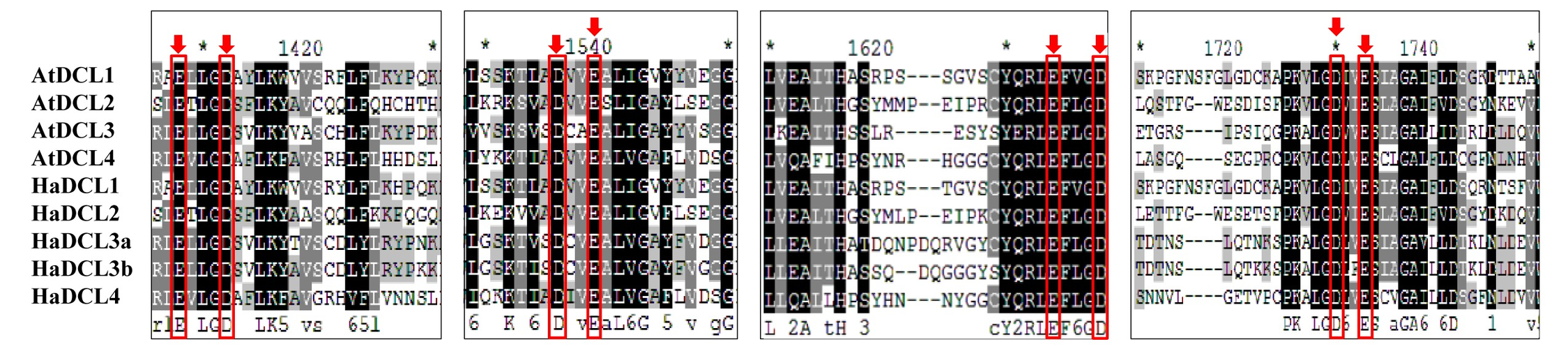

Supplement: S1 Fig — The investigation included the RNase III domains (RIBOc I and II), the Piwi domain, and the RdRP conserved domain. The conserved positions of the two RNase III domains at the glutamate (E), aspartate (D), glutamate (E), aspartate (D) (EDDE) position are indicated by downward red arrows. (TIF) [file pone.0286994.s005.tif]

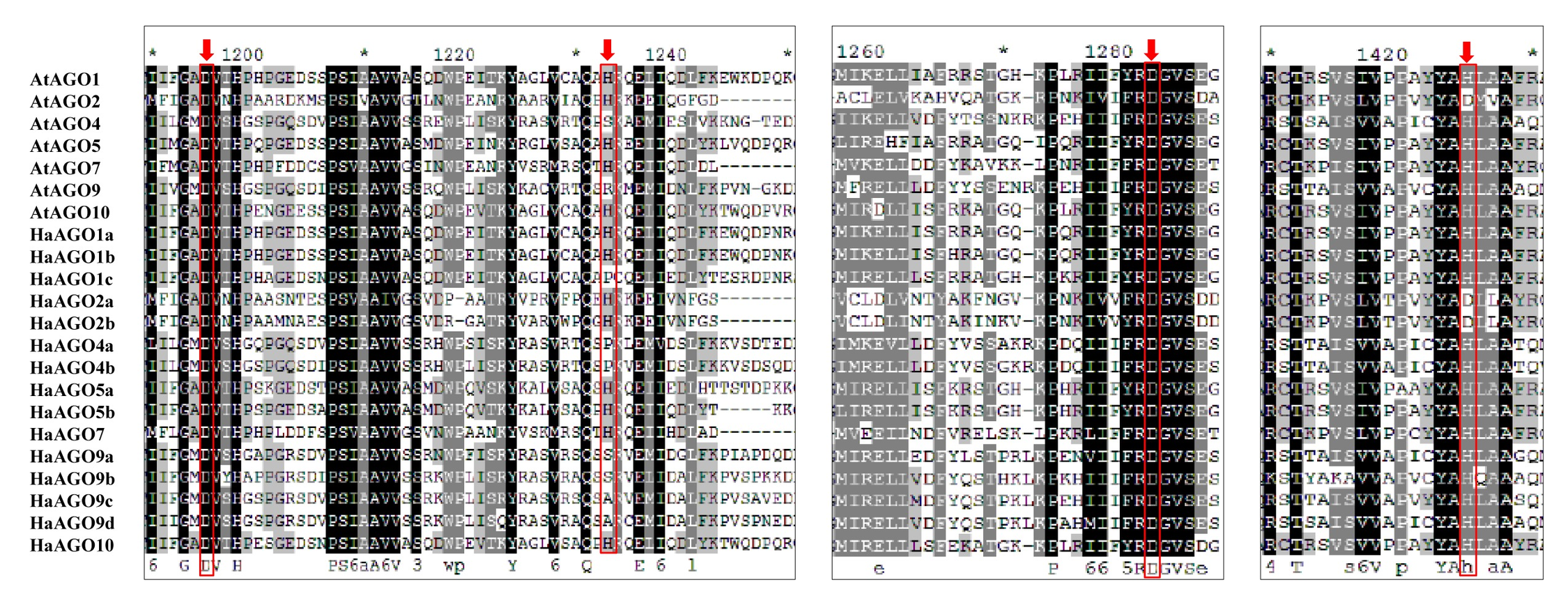

Supplement: S2 Fig — The conserved H798 locations and the conserved DDH triad of the Piwi domain are indicated by the descending red arrows. (TIF) [file pone.0286994.s006.tif]

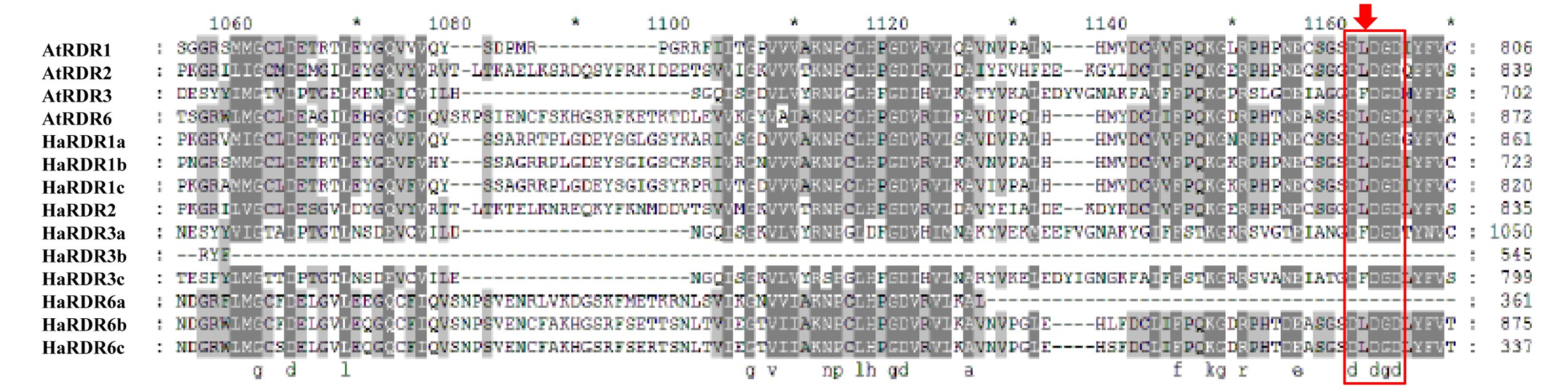

Supplement: S3 Fig — The red box indicates the conserved DxDGD catalytic motif. (TIF) [file pone.0286994.s007.tif]
